# A Challenging Picture of Cancer-and Inflammation-Related Changes

**DOI:** 10.4137/cmo.s2104

**Published:** 2009-03-02

**Authors:** Carsten Nieder, Rolf E. Steen, Astrid Dalhaug

**Affiliations:** 1 Medical Department, Division of Oncology, Nordlandssykehuset HF, Bodø, Norway.; 2 Faculty of Medicine, University of Northern Norway, Tromsø, Norway.; 3 Department of Pathology, Nordlandssykehuset HF, Bodø, Norway.

**Keywords:** lung cancer, head and neck cancer, abscess, metastasis, brain metastases

## Abstract

The authors describe a diagnostically challenging case where a patient with previous squamous cell carcinoma of the tonsil developed a putative second primary squamous cell carcinoma in the lung (stage IV with lung and bone metastases). During palliative chemotherapy several episodes of severe infection occurred, eventually resulting in abscess formation in the hip and brain. The dilemma of distinguishing between metastasis and abscess and the therapeutic implications are discussed.

## Case Description

**Figure 1 A–C.**
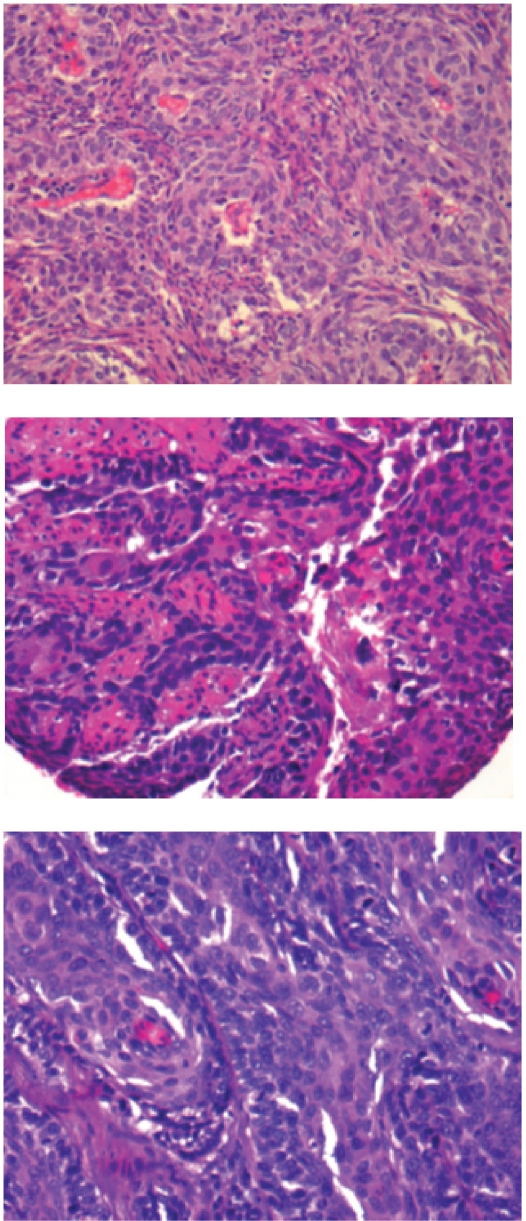
Histology slides (x40) of poorly differentiated squamous cell carcinoma in the tonsil, bronchial epithelium and skin.

A 61-year-old Caucasian male with a history of T2 N1 M0 squamous cell carcinoma of the tonsil (Fig. 1A), curatively treated with surgery and adjuvant radiation therapy six years earlier and with a history of continued smoking and insulin-dependent diabetes mellitus was admitted to the hospital for chest pain. After excluding acute cardiac changes, diagnostic work-up revealed a large mass in the upper lobe of the left lung as well as multiple small nodules in both lungs and multiple osteolytic bone changes, with no evidence of locoregional recurrence in the head and neck region or of enlarged intrathoracic lymph nodes. Histology of the lung mass derived from bronchoscopy showed poorly differentiated squamous cell carcinoma (Fig. 1B). No clear differentiation between metastases from the previous head and neck cancer and a second primary tumor could be made. Given the long time interval between the diagnosis of cancer the first time and the new findings with the evidence of tumor cells in the bronchial tree, a therapeutic approach for metastatic non-small cell lung cancer was chosen.

**Figure 2.**
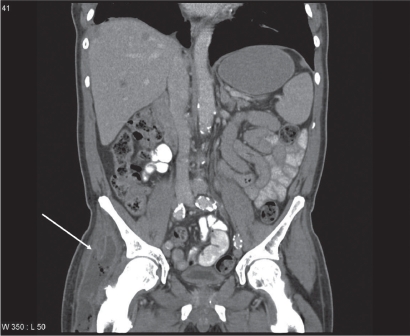
Contrast-enhanced computed tomography scan of the right hip after iv contrast injection: abscess indicated by white arrow.

**Figure 3.**
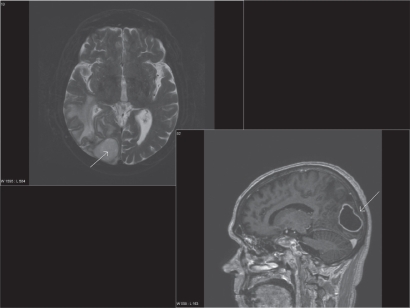
Axial T2 weighted and sagittal T1 weighted magnetic resonance image (MRI) after iv contrast administration: brain metastasis vs. abscess?

**Figure 4.**
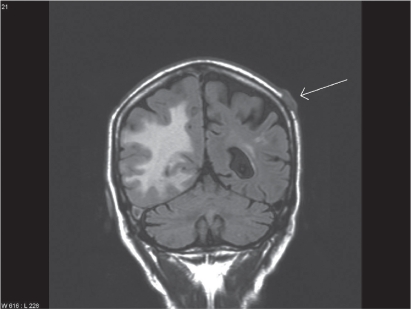
Different section of the MRI examination: cutaneous metastasis indicated by white arrow, note also the edema in the right hemisphere.

Treatment consisted of three courses of carboplatin/vinorelbine and monthly zoledronic acid. In the following period of stable disease (no reduction in lesion size), the patient developed three consecutive episodes of pneumonia, each accompanied by elevated C-reactive protein (CRP) levels, and resulting in hospitalisation. During the third episode, almost one year after initial presentation of chest pain, detection of liver and adrenal gland metastases indicated disease progression. Therefore, second line treatment with pemetrexed was initiated. After eight treatments, restaging computed tomography showed stable disease, but was indicative of abscess formation around the right hip (Fig. 2). Distinctly elevated CRP levels were also present. Following drainage, systemic treatment with antibiotics was initiated, yet microbiological examination of the fluid revealed sterile cultures. Six weeks later, the patient developed impaired vision, eventually leading to magnetic resonance imaging (MRI) of the brain. Different sequences were performed: T1-weighted with and without iv contrast, T2-weighted, fluid attenuated inversion recovery (FLAIR) and diffusion-weighted. Two non-contiguous lesions were detected—a large cystic lesion in the right occipital lobe (Fig. 3) and a solid lesion in the subcutis (Fig. 4). The low apparent diffusion coefficient (ADC) ratio, peripheral contrast enhancement and T2 hypointensity rim prompted the radiologist to state in his report that occipital lobe abscess was at least as likely as metastasis. The other lesion in the subcutis corresponded to a non-ulcerated 2 cm large nodule by physical examination. During this period, CRP was normal. Given the patient’s history and diagnostic uncertainty, invasive characterisation of the occipital mass and excision of the subcutaneous lesion was attempted in order to differentiate between new metastases and non-malignant causes. The subcutaneous lesion was histologically confirmed as poorly differentiated squamous cell carcinoma (Fig. 1C). During stereotactic biopsy, the other lesion was found to be a brain abscess and 15 ml of puriform fluid were drained. Local and systemic antibiotic treatment was initiated. Moreover, this time, the Gram’s stain showed inflammatory cells, but no bacterial or fungal source of infection could be determined. Further fluid evaluations, e.g. for mycobacteria as potentially causative organisms were also negative. Two months later, imaging follow-up showed resolution of the occipital mass, consistent with the diagnosis of brain abscess.

## Discussion

Patients with head and neck cancer, especially those who continue smoking, have an elevated risk for second primary tumors, which might arise in the same area, such as the esophagus, lungs or other organs.^1^ In a large analysis of more than 99,000 patients, lung cancer was the most common second primary.^2^ One of the reasons for the lower number of second primaries arising in the head and neck area might be that radiation treatment of the initial cancer also eradicates microscopic foci of potential second primaries.^3^ Prognosis after diagnosis of a second primary is typically limited, e.g. 12 months median survival in the series by.^4^ If both, the original lesion and the lung lesions are diagnosed as squamous cell cancers, the question arises whether the new lesion represents metastatic disease or a true second primary. In the present case, the occurrence of a second primary was somewhat more likely, although the histology slides really looked identical. As both lung and bone metastases were present, no serious therapeutic dilemma resulted (platinum-based chemotherapy and bisphosphonates without local measures would have been appropriate irrespective of diagnosis). Although not performed in the present case, human papilloma virus (HPV) typing and Comparative Genomic Hybridisation (CGH) analysis can be used in this setting for the differentiation between second primary tumors versus metastasis.^5^ HPV typing can be assessed on formalin fixed paraffin embedded tissue.

The patient reported here suffered several infectious complications during treatment. Thus, the detection of the brain lesion did not automatically result in the diagnosis of “brain metastasis”, which of course is a common finding in patients with metastatic lung cancer and which typically results in referral for palliative radiation therapy, although surgical resection might also be considered in a subgroup of patients. Both, abscesses and metastases typically form at the grey-white matter junction of the brain, but abscess is often related to adjacent localized cranial infection.^6^ In both conditions, MRI more often detects multiple than a solitary lesion.^7^,^6^ As recently discussed, information on ADC ratios and rim characteristics might help differentiate between abscess and neoplasm^8^ and also guided decision-making in the present case. Even in patients with known extracranial cancer metastases and a high risk for central nervous system involvement, newly detected brain lesions should not automatically be (mis)interpreted as dissemination from the malignant tumor.^9^,^10^ In immunocompromised patients and/or those with a recent history of infections, blood tests with or without examination of lesion fluid might provide valuable information towards appropriate management.^11^ Surgical aspiration and antibiotics should cure at least 90% of intracranial abscesses,^6^ while the prognosis of patients with brain metastases from lung cancer is poor despite the management options available today.^12^
